# Reaching Diverse Participants Utilizing a Diverse Delivery Infrastructure: A Replication Study

**DOI:** 10.3389/fpubh.2015.00077

**Published:** 2015-04-27

**Authors:** Matthew Lee Smith, Marcia G. Ory, SangNam Ahn, Basia Belza, Chivon A. Mingo, Samuel D. Towne, Mary Altpeter

**Affiliations:** ^1^Department of Health Promotion and Behavior, College of Public Health, The University of Georgia, Athens, GA, USA; ^2^Department of Health Promotion and Community Health Sciences, School of Public Health, Texas A&M Health Science Center, College Station, TX, USA; ^3^Division of Health Systems Management and Policy, School of Public Health, The University of Memphis, Memphis, TN, USA; ^4^Health Promotion Research Center, School of Nursing and School of Public Health, University of Washington, Seattle, WA, USA; ^5^Gerontology Institute, College of Arts and Sciences, Georgia State University, Atlanta, GA, USA; ^6^Center for Health Promotion and Disease Prevention, University of North Carolina at Chapel Hill, Chapel Hill, NC, USA

**Keywords:** chronic disease self-management, evidence-based program, participant reach, program implementation

## Abstract

This replication study examines participant recruitment and program adoption aspects of disease self-management programs by delivery site types. Data were analyzed from 58,526 adults collected during a national dissemination of the Stanford suite of chronic disease self-management education programs spanning 45 states, the District of Columbia, and Puerto Rico. Participant data were analyzed using multinomial logistic regression to generate profiles by delivery site type. Profiles were created for the five leading delivery site types, which included senior centers or area agencies on aging, residential facilities, healthcare organizations, community or multi-purpose centers, and faith-based organizations. Significant variation in neighborhood characteristics (e.g., rurality, median household income, percent of the population age 65 years and older, percent of the population i.e., non-Hispanic white) and participant characteristics (e.g., age, sex, ethnicity, race, rurality) were observed by delivery site type. Study findings confirm that these evidence-based programs are capable of reaching large numbers of diverse participants through the aging services network. Given the importance of participant reach and program adoption to the success of translational research dissemination initiatives, these findings can assist program deliverers to create strategic plans to engage community partners to diversify their participant base.

## Introduction

The grand-scale dissemination of evidence-based programs in community settings is contingent upon the presence of a delivery infrastructure capable of serving a large and diverse set of participants. Developing and nurturing the delivery infrastructure is important to ensure a reliable and sustainable community presence. Thus, in practice, community partners are encouraged to utilize many different delivery site types to ensure programs are available across geographic space. This ensures a greater likelihood that programs are offered close to participants’ residences in familiar settings that are easy to access (1, 2).

The types of agencies and organizations that adopt and deliver evidence-based programs can influence the types of participants reached. As postulated by the RE-AIM Framework (3, 4), program adoption and participant reach are closely related because a larger number of participants can participate in a program if more organizations adopt it and deliver workshops across a particular community or service region. Because certain organizations and delivery sites typically serve constituents with varying characteristics (e.g., age, sex, race/ethnicity), diversifying the types of delivery sites offering workshops has potential to increase diversity among evidence-based program participants (1). Further, people are more likely to enroll in programs/services that are in closer proximity to their residence. Thus, expanding the number of engaged delivery sites spanning the geographic service region may increase participant enrollment and program completion (i.e., increase attendance to ensure adequate intervention dose is received) (1).

This important issue was first examined in a study using data from the Administration on Aging (AoA)’s translation of the Evidence-Based Disease and Disability Prevention (EBDDP) program collected through the aging services network in community-based settings (1). This federal funding for evidence-based programs facilitated the delivery of Chronic Disease Self-Management Program (CDSMP) in 27 States from 2006 to 2009, which resulted in the development of a delivery infrastructure for evidence-based programs to serve older adults in various community-based settings. The AoA led the EBDDP initiative in partnership with the Centers for Disease Control and Prevention (CDC), Agency for Healthcare Research & Quality (AHRQ), Centers for Medicare and Medicaid Services (CMS), Health Resources & Services Administration (HRSA), Substance Abuse & Mental Health Services Administration (SAMHSA), and over 30 private foundations (1).

Findings from the initial study indicated that different delivery sites served areas with different neighborhood-level characteristics and participants with different personal and neighborhood-level characteristics (1). More specifically, the initial study found that, relative to workshops delivered at senior centers/area agencies on aging (AAA), the other delivery sites (i.e., residential facilities, healthcare organizations, community or multi-purpose centers, and faith-based organizations) were less likely to be offered in rural areas. Workshops delivered at healthcare organizations, community or multi-purpose centers, and faith-based organizations were more likely to be in more affluent areas. And, workshops in residential facilities and faith-based organizations were offered in areas with more non-Hispanic White residents compared to those offered in senior centers/AAA and community or multi-purpose centers. In terms of participant characteristics, relative to workshops delivered at senior centers/AAA, healthcare organizations, community or multi-purpose centers, and faith-based organizations were more likely to reach younger participants. Healthcare organizations and community or multi-purpose centers were more likely to reach male participants. Community or multi-purpose centers and faith-based organizations were more likely to reach African American participants, and healthcare facilities and faith-based organizations were more likely to reach participants residing in less affluent areas.

The aim of this replication study is to generate participant profiles by delivery site types to assess common and unique recruitment characteristics using chronic disease self-management education (CDSME) program data collected in 45 states, Puerto Rico, and the District of Columbia from 2010 to 2012. To replicate previous assessments with a more recent and expanded population frame (1), the purposes of this study were to: (1) describe CDSMP delivery site types in terms of their workshop and neighborhood-level characteristics; and (2) describe the personal and neighborhood-level characteristics of adults who enrolled in CDSME programs by delivery site type.

## Materials and Methods

### Program description

The CDSMP has been introduced and widely disseminated in the U.S. as a method to empower patients with self-management skills to deal with their chronic conditions (5). CDSMP is an evidence-based, peer-led intervention consisting of six highly participative classes held for 2.5 h each, once a week, for six consecutive weeks (5). CDSMP has resulted in improved healthcare and health (6, 7), while potentially saving healthcare costs (8). There is now a suite of CDSME programs licensed through the Stanford Patient Education Research Center, some of which are generic (e.g., CDSMP, Tomando Control de su Salud) and others that are disease specific (e.g., diabetes, arthritis, chronic pain). While the chronic condition may vary, all of these programs are based upon the social learning theory (9), highly interactive, and apply the principles of goal setting, problem solving, and action planning.

### Data source and study population

Cross-sectional data for this study were obtained from a nationwide delivery of CDSME programs as part of the American Recovery and Reinvestment Act of 2009 (i.e., Recovery Act) *Communities Putting Prevention to Work: Chronic Disease Self-Management Program* initiative (10). The U.S. Administration on Aging led this initiative in collaboration with the CDC and CMS to support the translation of CDSMP in 45 states, Puerto Rico, and the District of Columbia (11). This initiative was originally designed to have 50,000 Americans complete at least four out of six CDSMP sessions between 2010 and 2012 and to embed CDSMP delivery structures into statewide systems (10). For this study, data were analyzed from the first 100,000 participants who attended CDSMP workshops and had complete data for study variables of interest. As in the 2006–2009 initiative, systematic outcome data collection was not required because CDSMP is an evidence-based program. Thus, health-related outcomes are not reported in this study. Institutional Review Board approval for this study was obtained through Texas A&M University.

### Measures

#### Delivery Site Types

Data pertaining to CDSMP delivery site types were administratively collected (12). Only data from participants attending workshops in the five most prevalent delivery site types accounting for approximately 85% of classes were compared in these analyses: senior centers or AAA, residential facilities, healthcare organizations, community or multi-purpose centers, and faith-based organizations. These five delivery site types were consistent with those included in the previous study (1).

#### Neighborhood Characteristics

Using participants’ residential ZIP Codes, geographic information system (GIS) software was used to generate neighborhood-level variables for each participant. Neighborhood characteristics included residential rurality (i.e., metro residence or non-metro residence based on the rural-urban commuting area codes [RUCA]), median household income for residents residing in the participants’ ZIP Code (i.e., interpreted in increments of $10,000), the percent of residents aged 65 years and older residing in the participants’ ZIP Code, and the percent of non-Hispanic White residents residing in the participants’ ZIP Code (13). Using organizational ZIP Codes, GIS software was used to generate neighborhood-level variables for each delivery site (i.e., site rurality, median household income, percent of residents aged 65 years and older, and percent of non-Hispanic White participants).

#### Personal Characteristics

Personal characteristics of the participants included age, sex, race (i.e., non-Hispanic White, African American, Asian or Pacific Islander, American Indian or Alaska Native, Other/multiple races), and ethnicity (i.e., Hispanic, non-Hispanic). Participants also self-reported their living situation (i.e., lived alone, lived with others).

### Analyses

All statistical analyses were performed using SPSS (version 22). Of the first 100,000 participants reached in this initiative, cases were immediately omitted for those who attended workshops hosted at delivery sites other than the five most prevalent sites noted above (*n* = 13,784). The following delivery site types were omitted from analyses: educational institutions (*n* = 2,264, 2.3%), county health departments (*n* = 1,274, 1.3%), workplaces (*n* = 541, 0.5%), and tribal organizations (*n* = 189; 0.2%). Further, delivery sites categorized as “other” (*n* = 9,516, 9.5%) were omitted because of the potential difficulty to interpret findings associated with this delivery site type. Of the remaining 86,216 cases, those with missing data for age (*n* = 9,502), sex (*n* = 6,487), race (*n* = 14,278), ethnicity (*n* = 18,154), living situation (*n* = 60), residential rurality (*n* = 10,195), and delivery site rurality (*n* = 48) were subsequently omitted. Some participants had more than one of these exclusionary characteristics, thus the usable final sample was 58,526 middle-aged and older adults who attended CDSMP workshops at senior centers or AAA, residential facilities, healthcare organizations, community or multi-purpose centers, and faith-based organizations.

Frequencies were calculated for all major study variables, which were examined in relationship to the program delivery site type. Differences for categorical variables were assessed using Pearson’s chi-squared tests. One-way analyses of variance (*f* statistics) were used to identify mean differences for continuous variables. Multinomial logistic regression was used to examine personal characteristics and participants’ neighborhood-level characteristics associated with the type of delivery site they attended (i.e., senior center or AAA sites served as the referent group). Odds ratios and 95% confidence intervals are reported.

## Results

### Neighborhood characteristics by delivery site type

Neighborhood characteristics of the delivery site types are presented in Table 1. Of the 58,526 participants included in this study, 36.5% attended workshops at senior centers or AAA, 21.5% at residential facilities, 19.8% at healthcare organizations, 12.1% at community or multi-purpose centers, and 10.2% at faith-based organizations. Seventy-nine percent of participants attended workshops delivered in metro areas. On average, participants attended workshops delivered in ZIP Codes where the median household income was $50,400 (±$13,070) and in areas where 13.9% (±5.6%) of the population was aged 65 years and older. On average, participants attended workshops delivered in ZIP Codes comprised of 69.0% (±25.4%) non-Hispanic White residents.

**Table 1:** **Sample characteristics by delivery site type**.

	Total (*n* = 58,526)	Senior center/AAA (*n* = 21,339)	Residential facility (*n* = 12,600)	Healthcare organization (*n* = 11,577)	Community or multi-purpose facility (*n* = 7,068)	Faith-based organization (*n* = 5,942)	χ^2^ or *f*	*P*
**DELIVERY SITE CHARACTERISTICS**								
Metro (delivery site)	79.0%	76.9%	84.3%	76.4%	81.0%	78.4%	331.39	<0.001
Non-metro (delivery site)	21.0%	23.1%	15.7%	23.6%	19.0%	21.6%		
Median household income for ZIP code (delivery site)	50.40 (±13.07)	48.97 (±13.26)	51.91 (±12.69)	50.82 (±12.51)	51.07 (±13.17)	50.75 (±13.64)	115.39	<0.001
Percent of ZIP code population aged 65+ (delivery site)	13.93 (±5.59)	13.97 (±5.39)	14.20 (±5.59)	13.51 (±4.80)	13.96 (±6.33)	13.97 (±6.63)	24.43	<0.001
Percent of ZIP code population non-Hispanic White (delivery site)	68.95 (±25.35)	69.64 (±25.06)	67.33 (±26.67)	72.96 (±22.24)	65.83 (±26.53)	65.84 (±26.56)	139.84	<0.001
**PARTICIPANT CHARACTERISTICS**								
Age	68.58 (±13.56)	70.97 (±11.79)	73.44 (±12.81)	61.77 (±14.40)	66.03 (±13.76)	66.03 (±13.56)	1545.28	<0.001
Male	20.6%	19.1%	17.1%	25.6%	21.9%	21.6%	311.83	<0.001
Female	79.4%	80.9%	82.9%	74.4%	78.1%	78.4%		
Non-Hispanic	90.2%	90.8%	92.7%	86.7%	89.7%	89.9%	256.58	<0.001
Hispanic	9.8%	9.2%	7.3%	13.3%	10.3%	10.1%		
Non-Hispanic White	67.1%	68.4%	68.0%	74.1%	61.6%	54.1%	2029.69	<0.001
African American	21.5%	22.3%	23.2%	13.6%	20.9%	31.6%		
Asian/Pacific islander	4.2%	3.0%	3.0%	2.5%	9.9%	7.6%		
American Indian/Alaska native	1.2%	1.1%	0.9%	2.0%	1.0%	0.9%		
Other/multiple Races	5.9%	5.2%	5.1%	7.9%	6.6%	5.8%		
Number of self-reported chronic conditions	2.13 (±0.98)	2.63 (±1.62)	2.77 (±1.69)	2.64 (±1.64)	2.46 (±1.62)	2.29 (±1.59)	101.77	<0.001
Lives with others	96.3%	96.7%	96.5%	95.8%	95.3%	96.5%	38.24	<0.001
Lives alone	3.7%	3.3%	3.5%	4.2%	4.7%	3.5%		
Metro (participant residence)	77.9%	74.9%	83.9%	74.8%	81.1%	78.3%	478.07	<0.001
Non-metro (participant residence)	22.1%	25.1%	16.1%	25.2%	18.9%	21.7%		
Median household income for ZIP code (participant residence)	50.60 (±13.17)	49.11 (±13.30)	52.01 (±12.68)	51.14 (±12.78)	51.51 (±13.50)	50.80 (±13.57)	118.32	<0.001
Percent of ZIP code population aged 65+ (participant residence)	14.13 (±5.72)	14.26 (±5.54)	14.14 (±5.70)	13.93 (±5.01)	14.10 (±6.14)	14.05 (±7.06)	6.81	<0.001
Percent of ZIP code population non-Hispanic White (participant residence)	70.45 (±25.66)	71.12 (±25.29)	67.57 (±26.73)	74.88 (±23.26)	68.68 (±26.43)	67.57 (±26.86)	158.09	<0.001

*Pearson’s chi-squared tests (χ^2^) were used to identify significant distribution differences across delivery site types*.

*One-way analyses of variance (*f* statistics) were used to identify mean differences for continuous variables across delivery site types*.

When comparing these neighborhood characteristics by delivery site type, a larger proportion of workshops in non-metro areas were delivered in healthcare organizations (23.6%), senior centers/AAA (23.1%), and faith-based organizations (21.6%) compared to community/multi-purpose centers (19.0%) and residential facilities (15.7%). Little variation was observed based on the average median household income of workshops by delivery site types (i.e., range from $48,970 to $51,910). On average, workshops at faith-based organizations (65.8%) and community/multi-purpose facilities (65.8% non-Hispanic White) were delivered in more racially/ethnically diverse areas compared to workshops offered at healthcare organizations (73.0% non-Hispanic White).

### Participant characteristics by delivery site type

Personal characteristics of study participants are also presented in Table 1. Overall, the average age of participants was 68.6 years (±13.6). The majority of participants was female (79.4%), non-Hispanic (90.2%), non-Hispanic White (67.1%), and lived with others (96.3%). Approximately 78% of participants resided in metro areas. On average, participants resided in ZIP Codes where the median household income was $50,600 (±$13,170) and in areas where 14.1% (±5.7%) of the population was age 65 years and older. On average, CDSMP participants resided in ZIP Codes comprised of 70.5% (±25.7%) non-Hispanic White residents.

When comparing participant characteristics by delivery site type, residential facilities (73.4 years ± 12.8) and senior centers or AAA (71.0 years ± 11.79) recruited the oldest participants, on average. The largest proportion of male participants was reached in healthcare organizations (25.6%), whereas the smallest proportion was reached in residential facilities (17.1%). The greatest ethnic participant diversity was observed among workshops delivered at healthcare organizations (13.3%), community/multi-purpose facilities (10.3%), and faith-based organizations (10.1%). Relative to all other delivery site types, a substantially larger proportion of African American participants attended workshops at faith-based organizations (31.6%). Substantially, larger proportions of Asian or Pacific Islander participants attended workshops at community/multi-purpose facilities (9.9%) and faith-based organizations (7.6%). The largest proportions of participants living alone attended workshops at community/multi-purpose facilities (4.7%) and healthcare organizations (4.2%). The largest proportions of participants residing in non-metro areas were reached in healthcare organizations (25.2%), senior centers/AAA (25.1%), and faith-based organizations (21.7%).

The average area-level (ZIP Code-level) median household income varied by delivery site type. Participants who attended workshops at senior centers or AAA ($49,110 ± $13,170) resided in the least affluent areas, whereas those who attended workshops at residential facilities resided in the most affluent areas ($52,010 ± $12,680). The average area-level (ZIP Code-level) race/ethnicity composition also varied. Participants who attended workshops at faith-based organizations (67.6% non-Hispanic White residents ± 26.9%) and residential facilities (67.6% non-Hispanic white residents ±26.7%) resided in the most racially/ethnically diverse areas.

### Delivery site type profiles by neighborhood characteristics

Because senior center or AAA delivery site types were used as the referent group for regression analyses, the descriptive profile for this delivery site type by neighborhood characteristics is provided here (see Table 1). Approximately 77% of participants attended workshops delivered in metro areas. On average, participants attended workshops delivered in ZIP Codes where the median household income was $48,970 (±$13,260) and in areas where 14.0% (±5.4%) of the population was aged 65 years and older. On average, participants attended workshops delivered in ZIP Codes comprised of 69.6% (±25.1%) non-Hispanic White residents.

Utilizing multinomial logistic regression, profiles for residential facilities, healthcare organizations, community or multi-purpose centers, and faith-based organizations based on site neighborhood characteristics are described below. The senior center or AAA delivery site types were used as the referent group (see Table 2).

**Table 2:** **Delivery site neighborhood characteristics associated with delivery site type**.

	Residential facility				Healthcare organization				Community or multi-purpose facility				Faith-based organization			
			95% CI				95% CI				95% CI				95% CI	
	OR	*P*	Lower	Upper	OR	*P*	Lower	Upper	OR	*P*	Lower	Upper	OR	*P*	Lower	Upper
Non-metro (delivery site)	0.718	<0.001	0.67	0.77	1.152	<0.001	1.08	1.23	0.941	0.113	0.87	1.02	1.121	0.004	1.04	1.21
Metro (delivery site)	1.000	–	–	–	1.000	–	–	–	1.000	–	–	–	1.000	–	–	–
Median household income for ZIP code (delivery site)	1.023	<0.001	1.02	1.03	0.969	<0.001	0.96	0.97	1.016	<0.001	1.01	1.02	1.014	<0.001	1.01	1.02
Percent of ZIP code population aged 65+ (delivery site)	1.000	<0.001	1.00	1.00	1.000	<0.001	1.00	1.00	1.000	<0.001	1.00	1.00	1.000	<0.001	1.00	1.00
Percent of ZIP code population non-Hispanic White (delivery site)	0.996	<0.001	1.00	1.00	1.008	<0.001	1.01	1.01	0.993	<0.001	0.99	1.00	0.993	<0.001	0.99	0.99

*Referent group: senior center/AAA*.

*Significance: *P* < 0.001*.

*Odds ratios (OR) indicate the odds of a characteristic being associated with a delivery site type, relative to the referent group*.

#### Residential Facilities

Compared to workshops delivered at senior centers or AAA, participants were less likely to attend workshops delivered at residential facilities in rural areas (OR = 0.718, *P* < 0.001). Participants who attended workshops at residential facilities did so in areas that were more affluent (OR = 1.023, *P* < 0.001) and had smaller proportions of the population who were non-Hispanic White (OR = 0.996, *P* < 0.001).

#### Healthcare Organizations

Compared to workshops delivered at senior centers or AAA, participants were more likely to attend workshops delivered at healthcare organizations in rural areas (OR = 1.152, *P* < 0.001). Participants who attended workshops at healthcare organizations did so in areas that were less affluent (OR = 0.969, *P* < 0.001) and had larger proportions of the population who were non-Hispanic White (OR = 1.008, *P* < 0.001).

#### Community/Multi-Purpose Centers

Compared to workshops delivered at senior centers or AAA, participants who attended workshops at community or multi-purpose centers did so in areas that were more affluent (OR = 1.016, *P* < 0.001) and had smaller proportions of the population who were non-Hispanic White (OR = 0.993, *P* < 0.001).

#### Faith-Based Organizations

Compared to workshops delivered at senior centers or AAA, participants were more likely to attend workshops delivered at healthcare organizations in rural areas (OR = 1.121, *P* < 0.001). Participants who attended workshops at faith-based organizations did so in areas that were more affluent (OR = 1.014, *P* < 0.001) and had smaller proportions of the population who were non-Hispanic White (OR = 0.993, *P* < 0.001).

### Delivery site profiles by personal characteristics and residential characteristics

Because senior center or AAA delivery site types were used as the referent group for regression analyses, the descriptive profile for this site type by participant characteristics and their residential characteristics is provided here (see Table 1). The average age of participants was 71.0 years (±11.79). The majority of participants was female (80.9%), non-Hispanic (90.8%), non-Hispanic White (68.4%), and lived with others (96.7%). Approximately 75% of participants resided in metro areas. On average, participants resided in ZIP Codes where the median household income was $49,110 (±$13,300) and in areas where 14.3% (±5.5%) of the population was age 65 years and older. On average, CDSMP participants resided in ZIP Codes comprised of 71.1% (±25.3%) non-Hispanic White residents.

Utilizing multinomial logistic regression, profiles for residential facilities, healthcare organizations, community or multi-purpose centers, and faith-based organizations based on site neighborhood characteristics are described below. The senior center or AAA delivery site types were used as the referent group (see Table 3).

**Table 3:** **Personal characteristics associated with delivery site type**.

	Residential facility				Healthcare organization				Community or multi-purpose facility				Faith-based organization			
			95% CI				95% CI				95% CI				95% CI	
	OR	*P*	Lower	Upper	OR	*P*	Lower	Upper	OR	*P*	Lower	Upper	OR	*P*	Lower	Upper
Age	1.017	<0.001	1.02	1.02	0.951	<0.001	0.95	0.95	0.970	<0.001	0.97	0.97	0.971	<0.001	0.97	0.97
Female	1.126	<0.001	1.06	1.19	0.734	<0.001	0.69	0.78	0.921	0.016	0.86	0.99	0.896	0.003	0.83	0.96
Male	1.000	–	–	–	1.000	–	–	–	1.000	–	–	–	1.000	–	–	–
Hispanic	0.771	<0.001	0.70	0.85	1.157	0.001	1.06	1.26	1.006	0.915	0.91	1.12	1.233	<0.001	1.10	1.38
Non-Hispanic	1.000	–	–	–	1.000	–	–	–	1.000	–	–	–	1.000	–	–	–
Other/multiple races	1.046	0.436	0.93	1.17	0.981	0.726	0.88	1.09	1.119	0.084	0.99	1.27	1.118	0.125	0.97	1.29
American Indian/Alaska native	0.854	0.184	0.68	1.08	1.423	<0.001	1.17	1.73	0.926	0.577	0.71	1.21	0.968	0.832	0.71	1.31
Asian/Pacific islander	0.714	<0.001	0.62	0.82	0.675	<0.001	0.58	0.78	3.040	<0.001	2.70	3.43	3.044	<0.001	2.66	3.48
African American	0.816	<0.001	0.76	0.88	0.603	<0.001	0.56	0.65	0.913	0.039	0.84	1.00	1.951	<0.001	1.79	2.13
Non-Hispanic White	1.000	–	–	–	1.000	–	–	–	1.000	–	–	–	1.000	–	–	–
Lives alone	1.109	0.099	0.98	1.25	1.270	<0.001	1.12	1.44	1.575	<0.001	1.38	1.80	1.115	0.181	0.95	1.31
Lives with others	1.000	–	–	–	1.000	–	–	–	1.000	–	–	–	1.000	–	–	–
Non-metro (participant residence)	0.691	<0.001	0.65	0.74	1.047	0.154	0.98	1.11	0.787	<0.001	0.73	0.85	0.988	0.765	0.91	1.07
Metro (participant residence)	1.000	–	–	–	1.000	–	–	–	1.000	–	–	–	1.000	–	–	–
Median household income for ZIP code (participant residence)	1.000	<0.001	1.00	1.00	1.000	<0.001	1.00	1.00	1.000	<0.001	1.00	1.00	1.000	<0.001	1.00	1.00
Percent of ZIP code population aged 65+ (participant residence)	1.009	<0.001	1.01	1.01	0.999	0.629	0.99	1.00	1.018	<0.001	1.01	1.02	1.015	<0.001	1.01	1.02
Percent of ZIP code population non-Hispanic White (participant residence)	0.993	<0.001	0.99	0.99	1.004	<0.001	1.00	1.01	0.998	0.002	1.00	1.00	1.002	0.002	1.00	1.00

*Referent group: senior center/AAA*.

*Significance: *P* < 0.001*.

*Odds ratios (OR) indicate the odds of a characteristic being associated with a delivery site type, relative to the referent group*.

#### Residential Facilities

Compared to workshops attended at senior centers or AAA, participants who attended workshops delivered at residential facilities were more likely to be older (OR = 1.017, *P* < 0.001) and female (OR = 1.126, *P* < 0.001). These individuals were less likely to be Hispanic (OR = 0.771, *P* < 0.001) and less likely to be African American (OR = 0.816, *P* < 0.001), or Asian or Pacific Islander (OR = 0.714, *P* < 0.001). Participants who attended workshops delivered at residential facilities were less likely to reside in rural areas (OR = 0.691, *P* < 0.001). These participants also resided in areas that had larger proportions of the population who were age 65 and older (OR = 1.009, *P* < 0.001) and smaller proportions of the population who were non-Hispanic white (OR = 0.993, *P* < 0.001).

#### Healthcare Organizations

Compared to workshops attended at senior centers or AAA, participants who attended workshops delivered at healthcare organizations were less likely to be older (OR = 0.951, *P* < 0.001) and female (OR = 0.734, *P* < 0.001). These individuals were more likely to be American Indian or Alaska Native (OR = 1.423, *P* < 0.001), yet less likely to be African American (OR = 0.603, *P* < 0.001) or Asian or Pacific Islander (OR = 0.675, *P* < 0.001). Participants who attended workshops delivered at healthcare organizations were more likely to live alone (OR = 1.270, *P* < 0.001). These participants also resided in areas that had larger proportions of the population who were non-Hispanic White (OR = 1.004, *P* < 0.001).

#### Community/Multi-Purpose Centers

Compared to workshops attended at senior centers or AAA, participants who attended workshops delivered at community or multi-purpose centers were less likely to be older (OR = 0.970, *P* < 0.001). These individuals were more likely to be Asian or Pacific Islander (OR = 3.040, *P* < 0.001). Participants who attended workshops delivered at community or multi-purpose centers were more likely to live alone (OR = 1.575, *P* < 0.001) and less likely to reside in rural areas (OR = 0.787, *P* < 0.001). These participants also resided in areas that had larger proportions of the population who were age 65 and older (OR = 1.018, *P* < 0.001).

#### Faith-Based Organizations

Compared to workshops attended at senior centers or AAA, participants who attended workshops delivered at faith-based organizations were less likely to be older (OR = 0.971, *P* < 0.001). These individuals were more likely to be Hispanic (OR = 1.233, *P* < 0.001), African American (OR = 1.951, *P* < 0.001), or Asian or Pacific Islander (OR = 3.044, *P* < 0.001). Participants who attended workshops delivered at faith-based organizations resided in areas that had larger proportions of the population who were age 65 and older (OR = 1.015, *P* < 0.001).

## Discussion

Findings from this replication study support that CDSME programs are capable of attracting and serving a large and diverse group of participants using coordinated delivery infrastructure through the aging services network (1). In particular, the evidence-based programs delivered in the nationwide delivery of CDSME programs as part of the American Recovery and Reinvestment Act of 2009 initiative (12, 14, 15) reached many at-risk middle-aged and older adults in geographic regions of limited affluence and those with larger minority populations. Results indicate that certain delivery site types are more likely to serve geographic areas and participants with different characteristics, which highlights the importance of maintaining a diverse and dispersed collection of delivery sites in a given area/community to facilitate participants’ access to programs (16, 17). Other analyses of evidence-based programs for older adults reveal the mismatch between population needs and program availability (18). Thus, continued efforts are needed to recruit new community partners to establish and grow the existing infrastructure while simultaneously nurturing and supporting the existing infrastructure to ensure a sustained community presence (19).

This study is important in that it captures the continued growth and dispersion of CDSME programs from 2010 to 2012, the third wave of evidence-based health promotion/disease prevention programing supported by the Administration for Community Living (14, 15). The success of this intervention to reach over 100,000 participants in such a short time period is largely attributed to the previous success of the ACL-supported evidence-based program initiatives, which builds upon the infrastructure that was established from 2006 to 2009 (14). Continued monitoring of the reach of CDSME programs enables the visualization of the evolution of these programs as they are delivered throughout the United States. From 2006 to 2009, approximately 29,000 participants were reached across 27 states (17), while over 100,000 participants were reached across 45 states and two territories from 2010 to 2012. The leading five delivery site types remained consistent across these study periods, and senior centers and AAA consistently served the largest proportion of participants. However, there were some noteworthy changes in the areas served and the participants reached from 2006–2009 to 2010–2012. For example, in the 2010–2012 initiative, residential facilities emerged as delivery site types more likely to enroll females and participants residing in more affluent areas and areas with higher proportions of people aged 65 years and older compared to senior centers or AAA. Healthcare organizations emerged as delivery sites more likely to serve participants who reside alone and non-metro areas. Faith-based organizations emerged as delivery sites more likely to serve African Americans and Asian/Pacific Islanders. This reveals a greater diversification of delivery sites, and resonates with recommendations for capacity building and sustainability for institutionalizing programmatic activities (20).

In terms of translating the success of these evidence-based programs across multiple settings, the Consolidated Framework For Implementation Research (CFIR) has been highlighted for potential use (20). This framework pulls from, among other things, the idea that multiple theories can be combined to form a more comprehensive understanding of organizational characteristics associated with successful implementation of interventions (20). Future studies are encouraged to use this framework to identify organizational features associated with successful adaption and implementation of community-based programs by different delivery site types and programs. This would include the need to collect more comprehensive information about the delivery sites (e.g., culture, implementation climate). This information could also be used to develop targeted recommendations for organizations delivering these and other evidence-based programs (20).

This study reinforces the value of using the RE-AIM Framework when planning, implementing, and evaluating grand-scale translational initiatives to roll-out/disseminate evidence-based programs for older adults (21). More specifically, it supports the strong interdependence between program adoption and participant reach. However, this study did not examine the other important elements included in the RE-AIM Framework (e.g., implementation, evaluation, and maintenance), all of which are of equal importance for the success of grand-scale program dissemination.

There were limitations associated with this study. First, only limited data were collected from participants and delivery sites, which limited our ability to fully assess the characteristics of participants and sites that participated in this initiative. For example, outcomes were not collected for this grand-scale dissemination, thus it is unknown whether certain delivery site types evoked better health benefits than others and/or for which participants those benefits occurred. Second, there was considerable missing data for participant characteristics. This data collection issue was also observed in the initial study (1), and is likely attributed to self-report data collection occurring on-site and during workshop time at various locations across the country. Despite a coordinated data collection and reporting structure for this initiative, additional efforts may be needed to increase data fidelity as well as reduce data collection burdens on program deliverers. Third, while there were many statistically significant relationships observed in this study, such significant relationships may be an artifact of the large sample size and less about true differences across delivery site types. However, in an effort to be conservative, it should be noted that only relationships meeting the *P* < 0.001 criteria were deemed statistically significant for this study. Fourth, while this study provides insight about the reach and adoption elements of the RE-AIM Framework, additional investigations and data collection efforts are needed to understand the influence of this model on implementation, effectiveness, and maintenance in large translational evidence-based program dissemination efforts.

It should be noted that this study was not an exact replication of the earlier study (data from the years 2006 to 2009). The primary reason for differences was that the variables collected from 2006 to 2009 differed slightly from those collected from 2010 to 2012. For example, data related to participants’ education were not collected in the 2010–2012 initiative. Therefore, this variable could not be added to the analytic model. Among studies with older adults, education has been used as a proxy for social status because of issues related to self-reported household income (either based on missing data or that many older adults no longer work). Omitting education from the analyses reduced our ability to examine participant-level social status data; however, neighborhood-level data were utilized. Another example was that the categories of race/ethnicity differed between the studies. The data collected in the 2010–2012 initiative asked participants to report ethnicity separately and included more race categories (consistent with those in the U.S. Census) relative to the collapsed race/ethnicity item collected in the 2006–2009 initiative. While this change facilitated more nuanced analyses in the current study, it made direct race/ethnicity comparisons between studies more difficult.

A last example of replication differences was that the 2006–2009 initiative did not include participants’ self-reported chronic conditions. As such, the current study also omitted chronic conditions from the study analyses. However, because of the importance of participants’ chronic conditions for a disease self-management intervention, sensitivity analyses were performed that included self-reported chronic conditions in the participant-level multinomial regression model (tables not shown). On average, participants reported 2.60 (±1.64) chronic condition diagnoses, with 48.1% self-reporting three or more chronic conditions. All significant relationships remained significant in these sensitivity analyses. Relative to participants who attended workshops in senior centers or AAA, those who attended workshops in residential facilities and healthcare organizations reported significantly more chronic conditions; whereas those who attended workshops in community or multi-purpose centers or faith-based organizations reported significantly fewer chronic conditions.

## Conclusion

National replication studies are valuable for revealing the evolution of the infrastructure supporting evidence-based programs for older adults. Expanding upon current studies demonstrating the potential of CDSMP to meet the Triple Aims of health care reform (7), this replication’s findings suggest fertile areas for future study understanding about how delivery system characteristics are related to programmatic processes and outcomes. Additional research is needed to identify the most effective strategies for increasing organization-based recruitment efforts including both personal incentives and policies providing sustained support for CDSMPs for the increasingly diverse population of older Americans.

## Conflict of Interest Statement

The authors declare that the research was conducted in the absence of any commercial or financial relationships that could be construed as a potential conflict of interest.

*This paper is included in the Research Topic, “Evidence-Based Programming for Older Adults.” This Research Topic received partial funding from multiple government and private organizations/agencies; however, the views, findings, and conclusions in these articles are those of the authors and do not necessarily represent the official position of these organizations/agencies. All papers published in the Research Topic received peer review from members of the Frontiers in Public Health (Public Health Education and Promotion section) panel of Review Editors. Because this Research Topic represents work closely associated with a nationwide evidence-based movement in the US, many of the authors and/or Review Editors may have worked together previously in some fashion. Review Editors were purposively selected based on their expertise with evaluation and/or evidence-based programming for older adults. Review Editors were independent of named authors on any given article published in this volume*.
